# ‘Any and every cure for COVID-19’: an imminent epidemic of alternative remedies amidst the pandemic?

**DOI:** 10.11604/pamj.supp.2020.35.24728

**Published:** 2020-07-10

**Authors:** Ejemai Amaize Eboreime, Chinwe Juliana Iwu, Aduragbemi Banke-Thomas

**Affiliations:** 1 Department of Medicine, Faculty of Medicine and Dentistry, University of Alberta, Edmonton, Canada,; 2 Department of Nursing and Midwifery, Faculty of Medicine and Health Sciences, Stellenbosch University, Cape Town, South Africa,; 3 Department of Health Policy, London School of Economics and Political Science, London, United Kingdom

**Keywords:** COVID-19, epidemic, alternative medicine, health promotion, Africa

## Abstract

The magnitude of the COVID-19 pandemic is unprecedented, causing lots of apprehension among scientists, industry actors, politicians, and the general populace. Adverse health, social and economic effects of the pandemic have triggered an urgency among policy makers to seek an effective panacea. In this commentary, we examine the covert outbreak of a demand for alternative remedies with limited scientific evidence on their effectiveness to manage COVID-19 in Africa. Similar demands have been displayed in previous epidemics, though the ubiquity of social media in this current clime fuels such demands even more. We describe the attendant consequences of this demand surge on ongoing public health efforts to mitigate the spread of COVID-19 and highlight its future repercussions which may continue to plague health systems beyond the present outbreak. Going forward, governments must be proactive in surveillance of this covert epidemic, actively engage community influencers in knowledge transfer and implement targeted health promotion interventions.

## Correspondence

The unprecedented magnitude of the Coronavirus Disease (COVID-19) pandemic is significantly shifting the dynamics of the pharmaceutical industry, including the supply and demand for unorthodox remedies. The recent adverse health, social and economic effects of the ongoing pandemic have triggered a sense of urgency to seek an effective panacea by governments, with a ripple effect on health seeking behavior of the populace. Africa is the last continent to be affected by the outbreak. This gave ample time for the various governments, organizations like the African Union, and people to prepare ahead, learning from the experiences of other countries. Emergency operations centers were activated in various countries to coordinate response, laboratories were upgraded, public health communication strategies were designed, amongst other measures. But for a novel disease whose epidemiology and clinical characteristics are still being studied, no country can be prepared enough. Consequently, Africa has been battling to control the spread of the contagion since it arrived the continent early February 2020. However, another covert epidemic is brewing in Africa as a collateral effect of COVID-19, and the apprehension it has generated both within the leadership of African countries, and among its populace. This has to do with the abuse of untested ‘cures’, a ripple effect of the perceived confusion within the scientific community, and among political actors, as to which of the numerous proposed measures and therapies are truly effective. In this article, we examine the demand for alternative cures in Africa. We also warn about an imminent surge in abuse of presumed alternatives resulting from the ongoing political and social dynamics of the COVID-19 pandemic.

**Plurality of voices and alternatives from all and sundry:** the pressure to mitigate the morbidity and mortality of COVID-19, with the need to restore socioeconomic normalcy, has led to increased political interference in medical processes. For example, Donald Trump´s political pressure on regulatory bodies to accelerate approval of hydroxychloroquine (HCQ) for the treatment of COVID-19 [1]. This action influenced policy and individual behaviors worldwide, such that cases of HCQ toxicity following abuse were reported in faraway Nigeria. Even within the scientific community, controversies have arisen with validation of proposed therapies. Conflicting studies have been published regarding various prophylactic or treatment regimens such as the Bacille Calmette Guerin (BCG) vaccine and HCQ/macrolide combinations. These conflicts have triggered strong reactions among research and policy stakeholders, leading to the retraction of some studies with implications on decisions about ongoing clinical trials [2]. This urgency has not only affected the orthodox pharmaceutical market. Significant attention has also been drawn to traditional and complementary medicines (TCM), and other postulated non-pharmaceutical remedies. It is estimated that TCM is three to four times more commonly practiced than conventional medicine [3]. But the current dynamics will likely expand the TCM market further.

Recently, the president of Madagascar, Andry Rajoelina, announced a breakthrough herbal medicine for COVID-19 developed from the *Artemisia annua* by the Malagasy Institute of Applied Research [4]. COVID Organics, as the medication is branded, is said to possess prophylactic and curative properties. The announcement has been greeted by massive demand of this ‘cure’ by several African leaders. Heated debates across the world have also been elicited. Some argue that COVID Organics is scientifically untested for efficacy and safety. Others presume that, being herbal, the COVID organics is safe. But unknown to most herbal medicine users, this presumption is a fallacy. Public political endorsements of COVID organics has triggered market surge for TCMs. Many ‘cures’ are being proposed without any evidence of quality, safety, and efficacy. Cameroon for example, is experiencing shortages of herbal medicine due to high demand [5]. In Ghana, an undercover British Broadcasting Corporation video report showed conmen and quacks exploiting unsuspecting users by selling fake and toxic substances as COVID-19 cures for as much as $25,000 [6].

**Increasing demand for alternative remedies and its attendant consequences:** in Africa, this increase in demand is not a new phenomenon. Previous epidemics triggered widespread demand for speculative therapies. During the 2014 West African Ebola virus disease (EVD) crisis, there was an ‘epidemic’ salt toxicity occasioned by rumors that ingesting or bathing with concentrated salt solutions could prevent or cure the disease. A recent survey of survivors of the outbreak revealed that over 70% resorted to self-medication, and almost half of them used TCMs for post-EVD care [7]. Also, during the 1918 Spanish flu pandemic, the abuse of various speculative therapies across Africa was rife, as people resorted to inhaling eucalyptus and camphor [8]. Likewise, the current COVID-19 crisis has been associated with a covert epidemic of ‘miracle cures’ such as ingestion of bleach. Social media platforms have fueled the rapid propagation of misinformation about such purported cures. Attendant psychosocial and medical adverse events have also been recorded [9].

Psychosocial untoward effects reported with the use of either tested or speculative preventive therapies include a false sense of immunity. The abuse of alcohol, cannabis sativa and other psychoactive substances believed to prevent or cure the contagion can further aggravate this ‘superhuman’ grandeur, leading to deliberate exposure or undermining of public health measures, such as social distancing and the use of facemasks [4]. In addition, serious medical complications could result from drug interactions or direct pharmacological actions on biological organs and systems. Effects of such interactions could be immediate, such as cardiac arrests, or delayed events like chronic renal or hepatic failures and cancers. Congenital complications could affect unborn children [10]. Such cases end up requiring management by health workers, many of who are already significantly overwhelmed. Should this covert epidemic remain unaddressed, repercussions may continue to plague health systems beyond the present outbreak. While health systems irrespective of region and income status will all be vulnerable to such long-term shocks, African health systems are particularly vulnerable. Already, despite the continent´s successful battles with outbreaks like Ebola, the socioeconomic impact continues to undermine efforts towards sustainable resilient and responsive health systems. It is therefore pertinent that this collateral consequence of COVID-19 is raised among priority issues as African leaders and other stakeholders continue to evolve response strategies to the pandemic.

## Conclusion

Going forward, governments must be proactive in surveillance for this covert epidemic, and other collateral effects of the pandemic. Regulatory systems must be enhanced to mitigate abuse of controlled substances. Further, clinical trials must be strongly encouraged for TCMs to provide evidence of safety and efficacy. Efforts must be intensified to bridge the gap between knowledge producers and consumers, particularly policy makers, community influencers, and the general populace. Such knowledge brokerage must be incorporated into awareness campaigns about COVID-19 through traditional public and social media platforms to dispel existing and emerging rumors. Targeted health promotion interventions that recognize the motivations of diverse sub-groups of the population to seek alternative cures will be critical in stemming the demand. This needs to be done while providing the best available evidence in a manner and language that the target populations understand and can relate with. Community and religious leaders must also be involved in this process, given their strong influence and the established relationship between TCM, culture and religion. These efforts will go a long way to mitigate unintended consequences of COVID-19 and accelerate the restoration of society to post-pandemic normalcy.
